# Acute glucose influx-induced mitochondrial hyperpolarization inactivates myosin phosphatase as a novel mechanism of vascular smooth muscle contraction

**DOI:** 10.1038/s41419-021-03462-9

**Published:** 2021-02-12

**Authors:** Jie Xu, Hongyan Yang, Lu Yang, Zhen Wang, Xinghua Qin, Jiaheng Zhou, Ling Dong, Jia Li, Minsheng Zhu, Xing Zhang, Feng Gao

**Affiliations:** 1 School of Aerospace Medicine, Fourth Military Medical University, Xi’an, 710032 China; 2 Department of Cardiology, 986th Hospital, Fourth Military Medical University, Xi’an, 710032 China; 3 School of Basic Medical Sciences, Fourth Military Medical University, Xi’an, 710032 China; 4 Model Animal Research Center, Nanjing University, Nanjing, 210061 China

**Keywords:** Cardiovascular biology, Mitochondria

## Abstract

It is well-established that long-term exposure of the vasculature to metabolic disturbances leads to abnormal vascular tone, while the physiological regulation of vascular tone upon acute metabolic challenge remains unknown. Here, we found that acute glucose challenge induced transient increases in blood pressure and vascular constriction in humans and mice. Ex vivo study in isolated thoracic aortas from mice showed that glucose-induced vascular constriction is dependent on glucose oxidation in vascular smooth muscle cells. Specifically, mitochondrial membrane potential (ΔΨm), an essential component in glucose oxidation, was increased along with glucose influx and positively regulated vascular smooth muscle tone. Mechanistically, mitochondrial hyperpolarization inhibited the activity of myosin light chain phosphatase (MLCP) in a Ca^2+^-independent manner through activation of Rho-associated kinase, leading to cell contraction. However, ΔΨm regulated smooth muscle tone independently of the small G protein RhoA, a major regulator of Rho-associated kinase signaling. Furthermore, myosin phosphatase target subunit 1 (MYPT1) was found to be a key molecule in mediating MLCP activity regulated by ΔΨm. ΔΨm positively phosphorylated MYPT1, and either knockdown or knockout of MYPT1 abolished the effects of glucose in stimulating smooth muscle contraction. In addition, smooth muscle-specific *Mypt1* knockout mice displayed blunted response to glucose challenge in blood pressure and vascular constriction and impaired clearance rate of circulating metabolites. These results suggested that glucose influx stimulates vascular smooth muscle contraction via mitochondrial hyperpolarization-inactivated myosin phosphatase, which represents a novel mechanism underlying vascular constriction and circulating metabolite clearance.

## Introduction

Blood pressure (BP), one of the most commonly measured clinical parameters and one of the most commonly used indicators for cardiovascular health, is determined mainly by the blood volume, cardiac output, and vascular resistance^1–3^. Among these factors, vascular resistance depends on a balance between contraction and relaxation of vascular smooth muscle cells (VSMCs)^4,5^. Besides mechanical stimuli, vascular smooth muscle tone is mainly regulated by vasoactive signals released from sympathetic nerves, endocrine organs, and parenchymal cells, including epinephrine, noradrenaline, angiotensin II, endothelin, NO, etc.^4–6^. Recent studies have shown that vascular smooth muscle tone is also regulated by metabolism^7–10^. It is well-established that long-term exposure of the vasculature to metabolic disturbances leads to abnormal vascular tone^7,8^, while the physiological regulation of vascular tone upon acute metabolic challenge remains unknown.

The most abundant metabolite used by VSMCs is glucose. However, different from striated muscle cells (e.g., cardiomyocytes and skeletal muscle cells), VSMCs exhibit unusually high rates of glycolysis even under well-oxygenated condition, relying on a large extent of ATP from glycolysis other than glucose oxidation to maintain their biological activity^11^. It is estimated that only 30% of the ATP supply comes from mitochondrial oxidation, and at least 90% of glycolysis resulting in lactate production^12^. In spite of the low contribution of mitochondria to the VSMC bioenergetics, several lines of evidence highlight the relevance of mitochondria in the regulation of VSMC function^13^. For example, mitochondria were reported to be involved in regulating VSMC contraction, proliferation, migration, and secretion; and mitochondrial dysfunction contributes to vascular pathologies such as atherosclerosis, stenosis, and hypertension^10,13–15^.

VSMC contraction is normally initiated by the increase of intracellular Ca^2+^ concentration, resulting in Ca^2+^/calmodulin-dependent activation of myosin light chain (MLC) kinase (MLCK). MLCK phosphorylates MLC, allowing myosin to bind to actin filaments and thereby developing force and/or shortening cells^4,5^. In addition, MLC can be dephosphorylated by MLC phosphatase (MLCP), leading to cell relaxation. MLCP, a heterotrimer, is consisted of a catalytic type 1 of phosphatase subunit (PP1cδ), a myosin phosphatase target subunit 1 (MYPT1), and a 20-kD subunit^5^. MYPT1, the central regulator of MLCP, is mainly regulated by the small G protein RhoA and its downstream target Rho-associated kinase (ROCK). ROCK phosphorylates MYPT1, leading to the inhibition of MLCP activity and the resultant enhancement of MLC phosphorylation^4^. The other independent pathway involved in the regulation of MLCP activity is protein kinase C-activated protein phosphatase-1 inhibitor of 17 kD (CPI-17)^16,17^. CPI-17 behaves in a similar manner as MYPT1, whose activation inhibits MLCP activity. Recent evidence has shown that MLCP plays an important role in the regulation of vascular tone in both vascular health and diseases^16,18^.

Here, we explored the effects of glucose influx on vascular tone in humans and mice and found that blood glucose elevation induced vascular constriction in vivo. Mechanistically, glucose oxidation induced VSMC contraction through mitochondrial hyperpolarization-inactivated MLCP. These findings couple mitochondrial energetics with cell contraction in vascular smooth muscles, suggesting a new regulatory mechanism of vascular tone.

## Materials and methods

### Study population

The study was approved by the Human Research Ethics Committee of Fourth Military Medical University. Eight adults (male, 25–35 years old) without high risks for development of or diagnosed cardiovascular diseases were recruited. Informed written consent was obtained from each participant before enrollment. Subject characteristics are shown in supplemental Table 1. Oral glucose challenge was performed after 12 h overnight fast. Two tests with a 5-day interval were carried out for each participant. After oral ingestion of glucose or mannitol (1.0 g/kg body weight, dissolved in 200 mL water) in less than 2 min, blood glucose, BP, and ultrasonic imaging were measured at 0, 30, 60, and 120 min after challenge. Glucose or mannitol was assigned randomly to each participant at the first test, and the other challenge was assigned in the second test. All parameters were detected with the participant in a supine position. BP was measured using standard mercury sphygmomanometers by experienced observers as described previously^19^. The average or the last of 2 consecutive readings was recorded. The investigator was blinded to the group allocation when detecting the parameters.

### Animal model

Animal experiments were performed according to the National Institutes of Health Guidelines for the Use of Laboratory Animals, and were approved by Fourth Military Medical University Committee on Animal Care. C57 mice (male, 8 weeks) were purchased from the Animal Research Center of Fourth Military Medical University (China). Mice were randomly divided into different groups. The investigator was blinded to the group allocation when detecting the parameters. Mice were administered an intraperitoneal injection of glucose (2 g/kg) for glucose challenge after 12 h overnight fast. Blood glucose, blood pressure, and ultrasonic imaging were determined at 0, 15, 30, 60, and 90 min after the glucose challenge. Mice were administered a hypodermic injection of insulin (0.5 U/kg) for insulin challenge after 12 h overnight fast. Blood glucose was detected at 0, 30, 60, 90, and 120 min post insulin challenge.

Mypt1^SMKO^ mice were generated, genotyped and raised as reported previously^18,20^. Briefly, the chimeric mice carrying floxed *Mypt1* were crossed with SMA-Cre mice to ablate *Mypt1* specifically in smooth muscle. Mice with 2 floxed *Mypt1* alleles and *SMA-Cre* (*Mypt1*^flox/flox^; *SMA-Cre*) were used as Mypt1^SMKO^ mice. Male Mypt1^SMKO^ mice and the corresponding WT mice (8 wk) were used for experiments.

### Ultrasonography

Ultrasonography was performed with the ACUSON Sequoia 512 ultrasound machine (Siemens, New Jersey, USA) for humans and the Vevo 2100 imaging system (VisualSonics, Toronto, Canada) for mice. Cardiac ultrasound was used to determine stroke volume and heart rate. Cardiac output was calculated by stroke volume × heart rate. Vascular ultrasound was used to determine the maximal diameter of a brachial artery or abdominal aorta during vascular relaxation. Blood glucose, brachial BP, and ultrasonic imaging were simultaneously detected in humans, while blood glucose, caudal BP, and ultrasonic imaging were parallelly detected in mice.

### Functional assessment of thoracic aorta

Mice were sacrificed and the thoracic aortas were carefully excised and placed in ice-cold physiological saline solution (in mM: NaCl 119, KCl 4.6, MgCl_2_ 1.2, CaCl_2_ 1.5, NaHCO_3_ 15, NaH_2_PO_4_ 1.2, and glucose 5.0) as described previously^21^. The fat and connective tissue around the blood vessels were removed. The thoracic aorta segments (1 mm) were mounted in a temperature-controlled (37 °C) myograph system (DMT 610 M, Danish Myo Technology, Denmark) which was perfused with PSS continuously gassed with a mixture of 5% CO_2_ and 95% O_2_ (pH 7.35–7.45). An optimal initial length was applied for 1 hour before experiments. Endothelium removal was achieved by mechanical abrasion. Thoracic aorta segments were precontracted with 0.1 µM phenylephrine (PE) to test the effects of different treatments (metabolites, ionophores, and others) on vascular tone. After precontraction, thoracic aortas were challenged with glucose (30 mM), glucose (10 mM) + insulin (10 nM), FCCP (1 μM), nigericin (1 μM), valinomycin (1 μM), monesin (1 μM), NH_4_^+^ (5 mM), Ch_3_COO^-^ (5 mM), thapsigargin (TG) (5 μM), or BAPTA-AM (10 μM), unless noted otherwise.

For measurement of vascular function of vasodilation, aorta segments were precontracted with PE (10 μM) before treatments with acetylcholine (ACh) and sodium nitroprusside (SNP). Vasodilation evoked by cumulative ACh (10^−9^ to 10^−5^) or SNP (10^−10^ to 10^−5^ M) was determined and the results were expressed as the percentage of PE-induced contractile force. For measurement of vasoconstriction, aorta segments were not precontracted with PE and cumulative PE (10^−9^ to 10^−5^ M) and angiotensin II (10^−11^ to 10^−7^ M) were used to evoke vasoconstriction.

### VSMCs isolation and culture

Primary VSMCs were isolated from mouse thoracic aortas and cultured in DMEM as described previously^22^. Briefly, WT or Mypt1^SMKO^ mice were anesthetized by sodium pentobarbital (200 mg/kg, i.p.). The thoracic aorta was dissected, and the adhering periadventitial tissue and endothelium were removed. After removing the adventitial layer with Collagenase I solution (Sigma, 1 mg/ml) for 10 min at 37 °C, the medial layer was minced into small pieces for second digestion with Collagenase I for 2 h. Isolated VSMCs were cultured in DMEM containing 5 mM glucose, 10% FBS, and 1% penicillin/streptomycin-glutamine. VSMC purity was assessed by staining of smooth muscle-specific α-actin antibody (Santa). VSMCs between passages 2 (P2) to P5 were used. After serum starvation for 24 h, VSMCs were challenged with glucose (30 mM), glucose (10 mM) + insulin (10 nM), FCCP (1 μM), nigericin (1 μM), NH_4_^+^ (5 mM) or Ch_3_COO^-^ (5 mM) for 10 min, unless noted otherwise. VSMCs were pretreated with α-cyano-4-hydroxycinnamic acid (α-CCA, 200 μM), FCCP (1 μM), nigericin (1 μM), TG (5 μM), BAPTA-AM (10 μM), Y27632 (1 μM), or Fasudil (10 μM) at 30 min before metabolic challenges.

### Small interfering RNA (siRNA) transfection

For gene silencing assay, siRNAs for RhoA and MYPT1 were designed and purchased from GenePharma (Shanghai, China). The sequences of the oligos are shown in supplemental Table 2. VSMCs were transfected with siRNA or negative control by Lipofectamine™ 2000 (Invitrogen) following the manufacturer’s instructions. The efficiency of gene knockdown was detected by western blot at 60 h after siRNA transfection.

### Confocal imaging

An inverted confocal microscope (Zeiss LSM 800, 40×, 1.3 NA oil-immersion objective) was used for imaging. Cells or blood vessels were incubated with Tyrode’s solution consisting of (in mM: NaCl 137, KCl 5.4, MgCl_2_ 1.2, NaH_2_PO_4_ 1.2, 1.0 CaCl_2_, glucose 5.0, and HEPES 20, pH 7.35–7.45). For measurement of ΔΨm, JC-1 (50 μM) or TMRM (20 nM) was loaded at 37 °C for 20 min followed by 3 times washing with Tyrode’s solution. To obtain JC-1 signals, images were captured by tandem excitation of scan-lines at 488 nm and 573 nm, and emission collection at 500–570 nm (monomer) and 600–670 nm (aggregate). The fluorescence ratio (monomer/aggregate) indicates the changes of ΔΨm. To obtain TMRM signals, images were captured by excitation at 573 nm and emission collection at 600–650 nm. All experiments were performed at room temperature (22–24 °C). For measurement of ΔΨm within the smooth muscle of intact thoracic aorta, the arteries were maintained at physiological pressure as described previously^14^.

### Western blot

Proteins were extracted from either blood vessels or VSMCs. The levels of protein expression or phosphorylation were measured using Western blot as described previously^23^. The immunoblots were probed with anti-p-MLC (Ser20), anti-MLC, anti-p-MYPT1 (Thr853), anti-MYPT1, anti-ROCK1, anti-ROCK2, anti-p-CPI-17 (Thr38), anti-CPI-17, anti-RhoA, and anti-β-actin antibodies (Abcam/Cell Signaling Technology) overnight at 4 °C followed by incubation with the corresponding secondary antibodies at room temperature for 1 h. RhoA activation was measured with the Rho Activation Assay Kit from Cell Biolabs according to the manufacturer’s instructions (STA-403-A).

### Circulating metabolite clearance

The mice were injected with a bolus of 2-NBDG (10 μg/g), a fluorescent-labeled glucose analogy, or indocyanine green (ICG) (10 μg/g), a low-toxic fluorescent that is selectively taken up by hepatocytes, via caudal vein (0.2 mL) after 12 h overnight fast. Meanwhile, glucose (2 g/kg) or saline was intraperitoneal injected. Blood samples were taken at 5, 30, 60, 90, and 120 min after injection. The 2-NBDG and ICG fluorescence in serum was determined spectrophotometrically at 488 and 800 nm, respectively.

### Statistical analysis

The sample size for the human study was estimated using a sample size calculator (www.calculator.net). Sample sizes for other experiments were chosen based on studies with similar experimental design and on the known variability of the assay. All values are presented as mean ± SEM. Data were compared with one-way ANOVA or two-way ANOVA, with all ANOVA tests followed by a paired (for human data) or unpaired (for other data) two-tailed t-test, as appropriate. Bonferroni’s correction for multiple comparisons was used. Linear relationships between variables were tested by Pearson’s correlation coefficient. Normal distribution of data was analyzed by Kolmogorov–Smirnov normality test. In all statistical comparisons, *p* value of less than 0.05 was considered to be statistically significant. No samples/animals were excluded, except excluding some immunofluorescent data prior to analysis due to techinical issues (out of focus, auto-fluorescent debris, etc).

## Results

### Glucose challenge induced vascular constriction in vivo

**Fig. 1:**
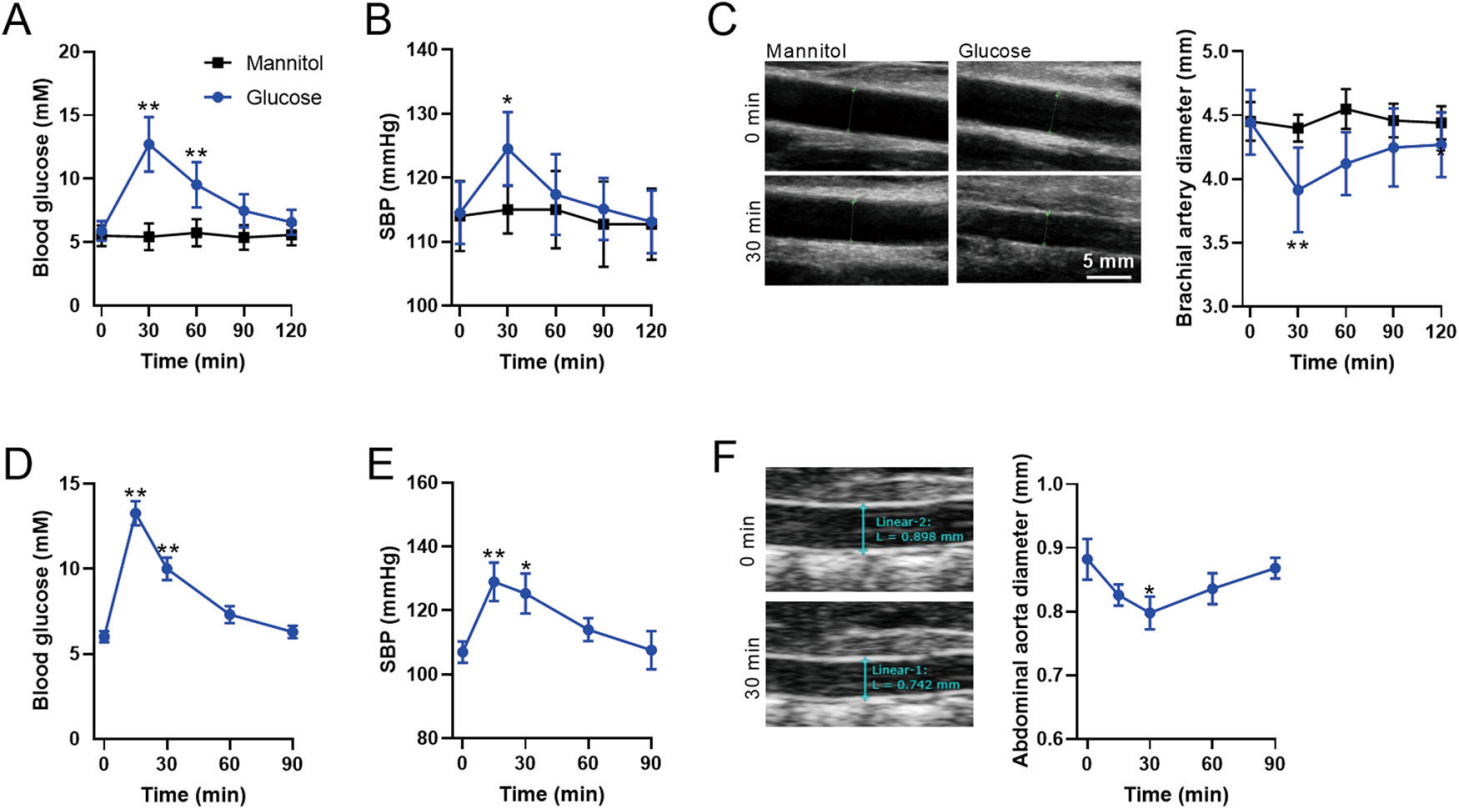
Glucose challenge induced vascular constriction in humans and mice. **A**–**F** Blood glucose (**A**), systolic blood pressure (SBP) (**B**) and the maximal brachial artery diameter (**C**) in response to oral glucose challenge in humans. Representative images of the maximal diameter of brachial artery in response to glucose challenge are shown in left, and the quantified results are shown in right (**C**). *n* = 8 subjects. **D**–**F** Blood glucose (**D**), SBP (**E**), and the maximal abdominal aorta diameter (**F**) in response to intraperitoneal glucose challenge in mice. Representative images of the maximal abdominal aorta in response to glucose challenge are shown in left, and the quantified results are shown in right (**F**). *n* = 8 mice. Error bars represent SEM. ^*^P < 0.05. ^**^P < 0.01.

To examine the effects of glucose influx on vascular tone, a total of 8 healthy adults (male, 25–35 years old) were recruited. Oral glucose challenge (1.0 g/kg body weight) resulted in a transient increase in blood glucose, reaching its peak level at 30 min and falling to euglycemia at 2 h post-challenge (Fig. 1A). Simultaneous measurement of BP and cardiac function showed that systolic BP was increased by 8.7% at 30 min post-challenge (Fig. 1B), while diastolic BP and cardiac output displayed no significant differences (supplemental Fig. 1), suggesting that an increase in systemic vascular resistance may underlie the evaluation of systolic BP. In fact, a decrease in the maximal diameter of the brachial artery was observed (decreased by 12.0%) at 30 min post challenge (Fig. 1C). There was a negative linear relationship between blood glucose and the maximal brachial artery diameter in subjects with glucose challenge (supplemental Fig. 1). Similarly, intraperitoneal glucose challenge (2 g/kg body weight) resulted in transient increases in blood glucose and systolic BP in mice (Fig. 1D, E). In addition, parallelly measurement of the maximal abdominal aorta diameter revealed that abdominal aorta diameter was decreased at 30 min post-challenge in mice (Fig. 1F). These results suggested that glucose challenge increases vascular constriction and systolic BP in both humans and mice.

### Glucose-induced vascular constriction is dependent on mitochondrial oxidation in VSMCs

**Fig. 2:**
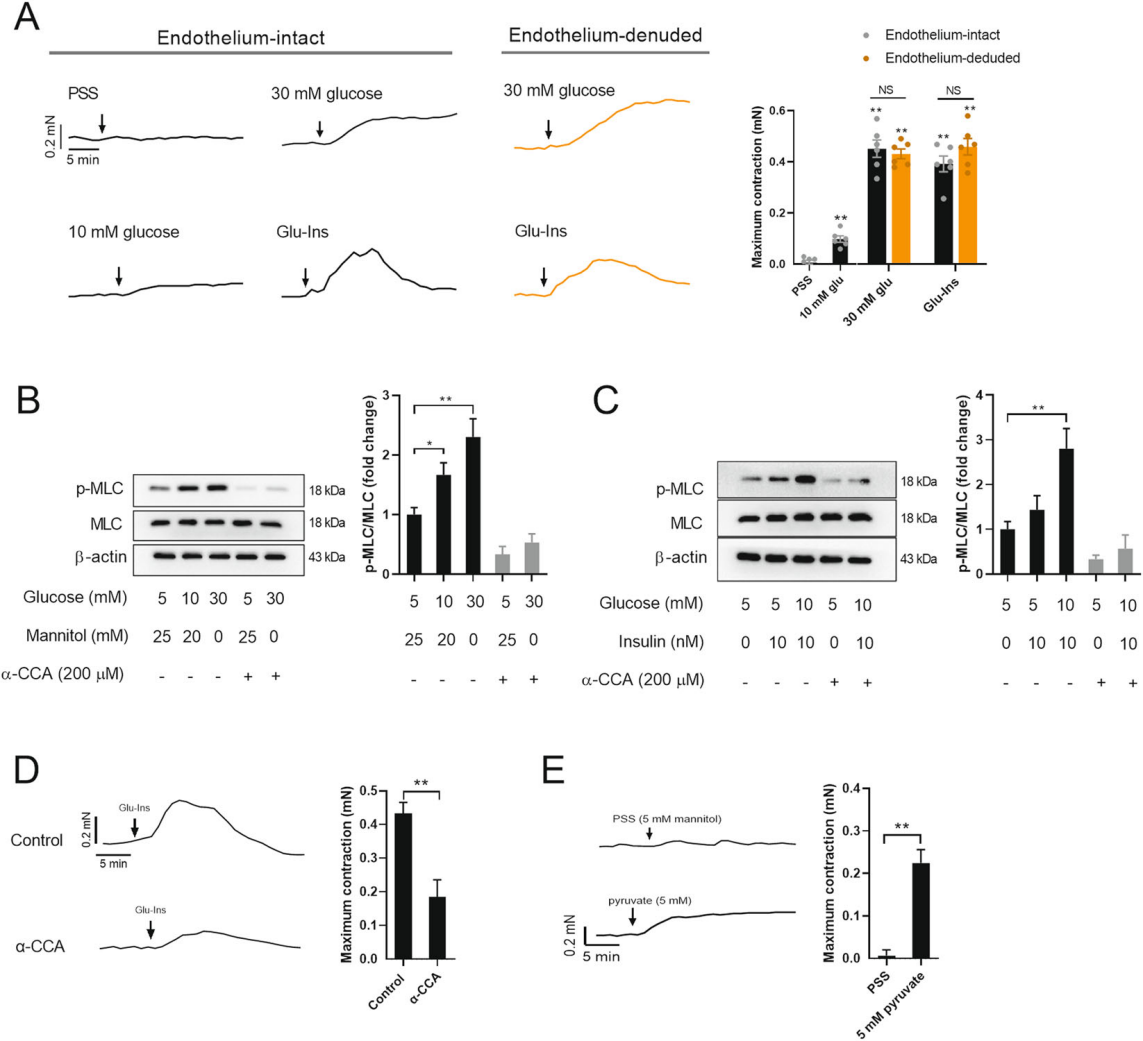
Glucose-induced vascular constriction is dependent on mitochondrial oxidation in VSMCs. **A** Glucose (10, 30 mM) and Glu-Ins (10 mM glucose +10 nM insulin) induced vascular constriction in isolated thoracic aortas with intact endothelium or with denuded endothelium. The aortas were pretreated with PE. Typical tension curves of thoracic aortas were shown in the left, and the quantified results were shown in right. Osmotic pressure was adjusted with mannitol. ^*^, vs. saline. *n* = 6. **B**, **C** Glucose (**B**) or Glu-Ins (**C**) induced MLC phosphorylation, which was blocked by α-CCA in cultured VSMCs. *n* = 5. **D** α-CCA pretreatment blocked the effects of Glu-Ins on induction of vascular constriction in isolated thoracic aortas. The aortas were pretreated with PE. *n* = 6. **E** Pyruvate induced vascular constriction in isolated thoracic aortas. The aortas were pretreated with PE. *n* = 6. Error bars represent SEM. ^*^P < 0.05. ^**^P < 0.01.

To test whether glucose induces vascular constriction directly, the effect of glucose on vascular constriction was determined ex vivo. As shown in Fig. 2A, glucose (10 and 30 mM) induced thoracic aorta constriction in a dose-dependent manner. To mimic the glucose challenge in vivo, a mixture of glucose and insulin (10 mM glucose and 10 nM insulin, Glu-Ins) was used. Glu-Ins induced vascular constriction to an extent similar to that of 30 mM glucose (Fig. 2A). However, Glu-Ins-induced vascular constriction was reversible, reaching its peak level at 12.2 ± 2.3 min and falling to baseline at 25.2 ± 4.5 min (Fig. 2A), probably due to a transient response of insulin (supplemental Fig. 2). On the contrary, glucose-induced vascular constriction was relatively stable and lasted at least for 60 min. These results suggested that glucose metabolism directly stimulates vascular constriction.

Endothelium removal showed no significant effect on glucose- or Glu-Ins-induced vascular constriction ex vivo, suggesting that glucose-induced vascular constriction is VSMC-dependent (Fig. 2A). As expected, glucose and Glu-Ins increased MLC phosphorylation in cultured VSMCs (Fig. 2B, C). Interestingly, both glucose- and Glu-Ins-induced MLC phosphorylation were blocked by α-CCA (200 μM), an inhibitor of the mitochondrial pyruvate transporter, suggesting that glucose oxidation is necessary in glucose-induced VSMC contraction (Fig. 2B, C). α-CCA pretreatment (200 μM) also blocked the effects of Glu-Ins on induction of vascular constriction in isolated thoracic aortas (Fig. 2D). In fact, glucose increased mitochondrial oxidation as evidenced by increased respiration (supplemental Fig. 3). Furthermore, pyruvate, one of the end products of glycolysis, induced vascular constriction in isolated thoracic aortas (Fig. 2E). These results suggested that glucose-induced vascular constriction is dependent on mitochondrial oxidation in VSMCs.

### Mitochondrial membrane potential positively regulates VSMC contraction

**Fig. 3:**
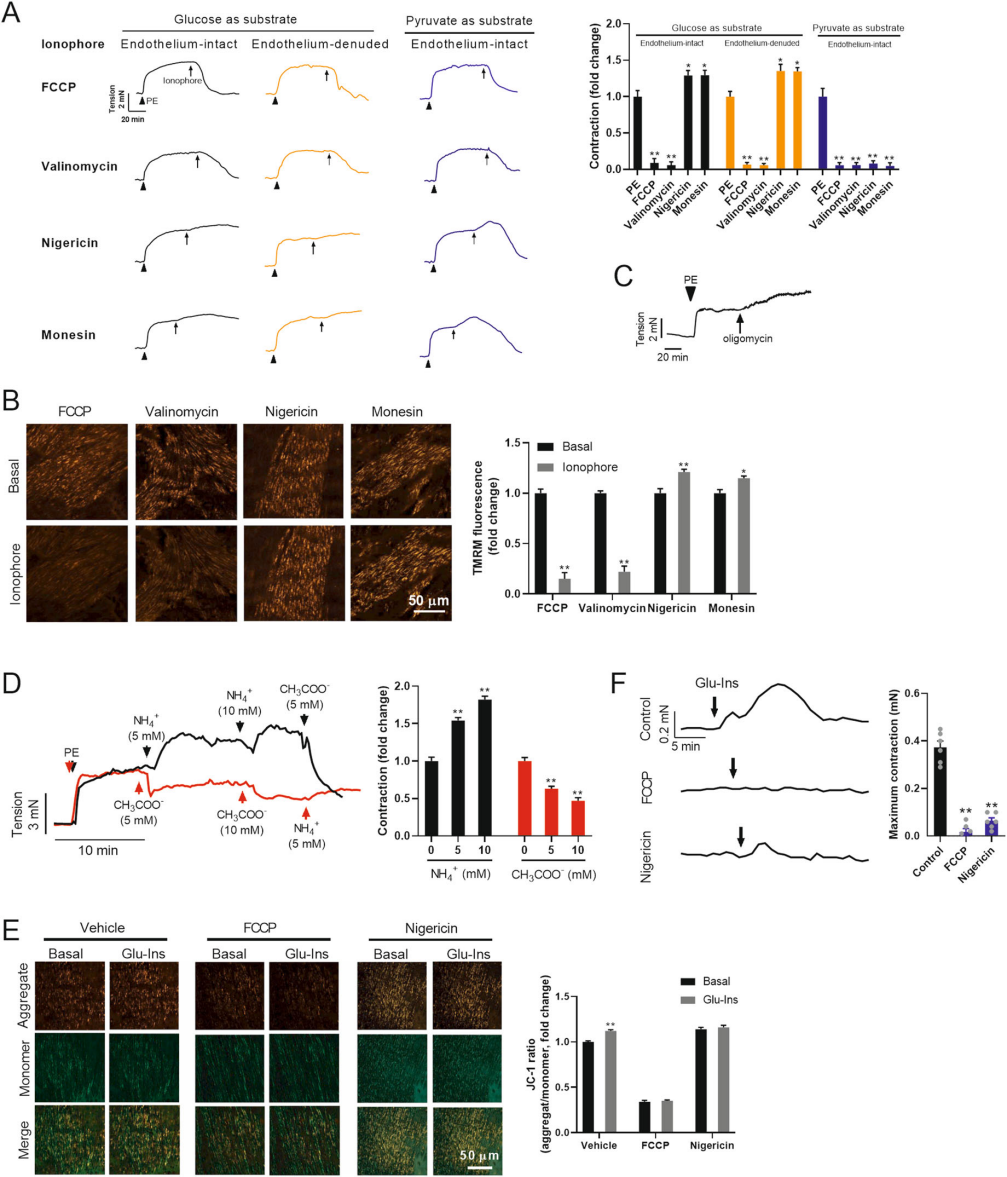
ΔΨ_m_ positively regulates VSMC contraction. **A** The effects of four ionophores on vascular constriction in isolated thoracic aortas with intact endothelium or with denuded endothelium. The aortas were pretreated with PE. Glucose (5 mM) or pyruvate (5 mM) was supplied as the only metabolite available in the incubation medium. Typical traces of vascular tension were shown in the left, and the quantified results were shown in right. The arrow indicates the time point for treatment with ionophore. *n* = 6. **B** The effects of four ionophores on ΔΨ_m_ in VSMCs of isolated aortas. Typical images of ΔΨ_m_ in VSMCs in blood vessels were shown in the left, and the quantified results were shown in right. ΔΨ_m_ was detected by TMRM. *n* = 6. **C** Oligomycin induced vascular constriction in isolated thoracic aortas. **D** The effects of NH^4+^ and CH_3_COO^-^ on vascular tone in isolated aortas. Typical traces of vascular tension were shown in the left, and the quantified results were shown in right. *n* = 6. **E** Glu-Ins-induced mitochondrial hyperpolarization was blocked by preincubation of isolated aortas with FCCP or nigericin. ΔΨ_m_ was detected by JC-1. Typical images of ΔΨ_m_ in VSMCs in blood vessels were shown in the left, and the quantified results were shown in right. **F** Preincubation of isolated aortas with FCCP or nigericin inhibited Glu-Ins-induced vascular constriction. Typical traces of vascular tension were shown in the left, and the quantified results were shown in right. *n* = 6. Error bars represent SEM. ^*^P < 0.05. ^**^P < 0.01.

In another set of experiments, we aimed to investigate which component in mitochondrial oxidation is involved in the regulation of VSMC contraction using four ionophores. FCCP, an electrical H^+^ ionophore, dissipating proton gradient (ΔpH) and eliminating ΔΨm and mitochondrial ATP production^24^. Valinomycin, an electrical K^+^ ionophore, depolarizes ΔΨm and eliminates mitochondrial ATP formation^24^. Nigericin, an electroneutral H^+^/K^+^ ionophore, eliminates ΔpH and mitochondrial ATP production and results in a compensating increase in ΔΨm^24^. Monesin, an electroneutral H^+^/Na^+^ ionophore, exerts the similar effects as that for nigericin. The results showed that FCCP and valinomycin disrupted vascular constriction to baseline, while nigericin and monensin increased vascular constriction in both endothelium-intact and endothelium-denuded aortas (Fig. 3A). These results indicated that ΔΨm is a potential regulator of VSMC contraction, while mitochondrial ATP formation is not necessary in the induction of VSMC contraction. The effects of these ionophores on ΔΨm in VSMCs were shown in Fig. 3B. To exclude non-specific effects of these ionophores on ΔΨm, oligomycin which blocks mitochondrial ATP production and increases ΔΨm was used. Oligomycin also increased vascular constriction in isolated thoracic aortas (Fig. 3C).

Although mitochondrial ATP formation is not necessary for mitochondrial hyperpolarization-induced vascular constriction, ATP from glycolysis is necessary as evidenced by that all these ionophores disrupted vascular constriction when pyruvate (5 mM) was supplied as the only metabolite in the incubation medium (Fig. 3A). To extend our findings, CH_3_COO^-^ (5 mM) and NH_4_^+^ (5 mM), classic tools in the intervention of ΔΨm, were employed. CH_3_COO^-^ depolarized ΔΨm and NH_4_^-^ hyperpolarized ΔΨm. As a result, CH_3_COO^-^ induced vascular relaxation and NH_4_^+^ promoted vascular constriction in a dose-dependent manner in isolated aortas (Fig. 3D). It should be noted that CH_3_COO^-^ and NH_4_^+^ also affect mitochondrial pH. These results reinforced the notion that ΔΨm positively regulates VSMC contraction.

### Mitochondrial hyperpolarization contributes to glucose-induced VSMC contraction

To test whether glucose-induced vascular constriction is dependent on mitochondrial hyperpolarization, FCCP and nigericin were used to clamp ΔΨm in isolated aortas and cultured VSMCs. Glu-Ins induced an increase in ΔΨm in VSMCs of isolated aortas (Fig. 3E), and preincubation of isolated aortas with either FCCP or nigericin inhibited the Glu-Ins-induced mitochondrial hyperpolarization (Fig. 3E). As a result, Glu-Ins-induced vascular constriction was inhibited in isolated aortas preincubated with FCCP or nigericin (Fig. 3F). In addition, preincubations of cultured VSMCs with FCCP or nigericin blocked the mitochondrial hyperpolarization, as well as MLC phosphorylation, induced by glucose (30 mM) (supplemental Fig. 4). These results suggested that mitochondrial hyperpolarization contributes to glucose-induced VSMC contraction.

### Glucose induces VSMC contraction through inhibition of MLCP

**Fig. 4:**
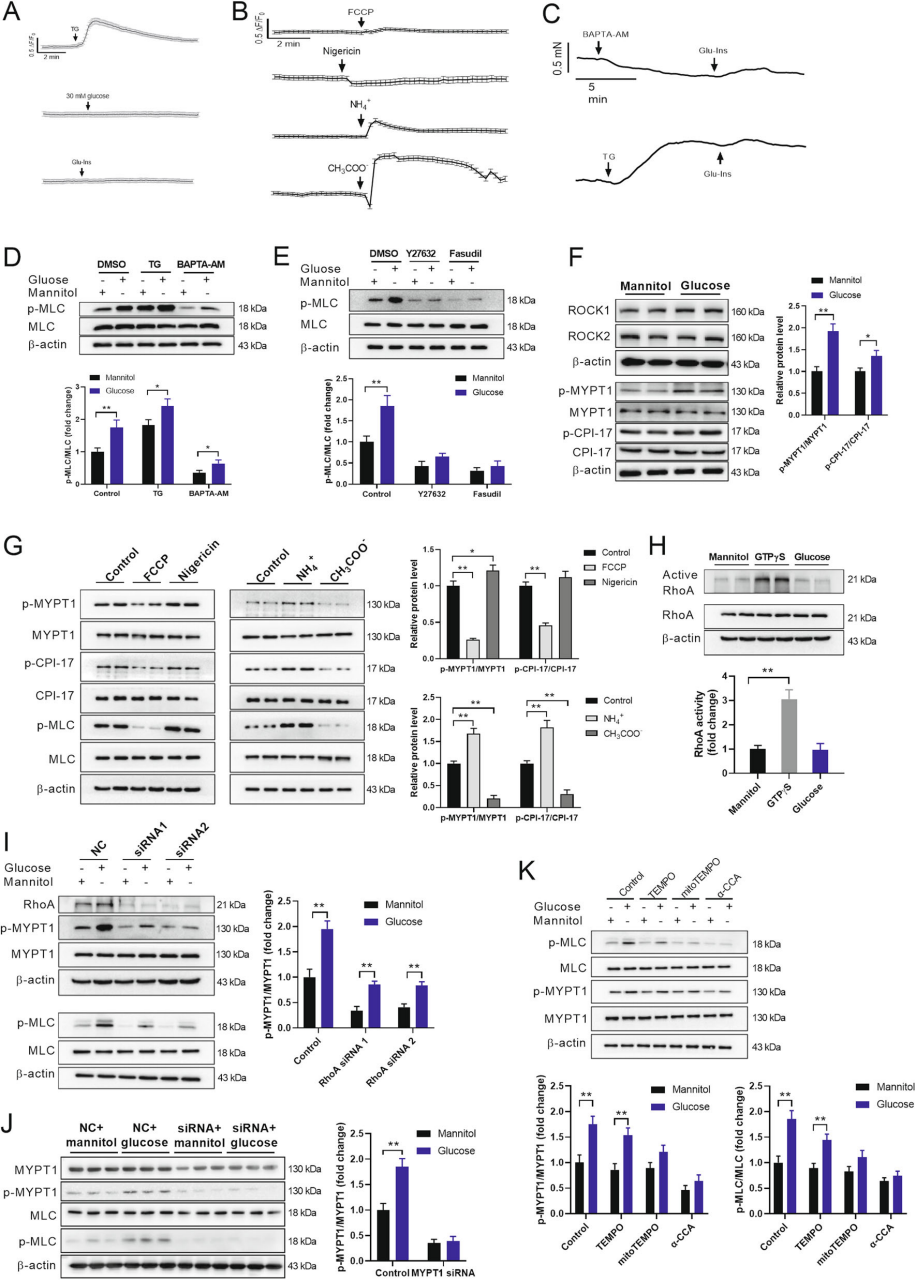
Glucose induces VSMC contraction through inhibition of MLCP. **A** The effects of glucose (30 mM) and Glu-Ins on intracellular Ca^2+^ in VSMCs. *n* = 5. **B** The effects of FCCP, nigericin, CH_3_COO^-^ and NH_4_^+^ on intracellular Ca^2+^ in VSMCs. *n* = 5. **C** The effects of Glu-Ins on vascular constriction in isolated aortas preincubated with either thapsigargin (TG) or BAPTA-AM. *n* = 5. **D** The effects of glucose (30 mM) on MLC phosphorylation in cultured VSMCs preincubated with either TG or BAPTA-AM. *n* = 5. **E** The effects of glucose (30 mM) on MLC phosphorylation in cultured VSMCs preincubated with either Y27632 or Fasudil. *n* = 5. **F** Glucose (30 mM) increased phosphorylation of MYPT1 and CPI-17 in cultured VSMCs. *n* = 5. **G** Nigericin and NH_4_^+^ increased MYPT1 phosphorylation and FCCP and CH_3_CHOO^-^ decreased MYPT1 phosphorylation in cultured VSMCs. *n* = 5. **H** Glucose (30 mM) showed little effects on both RhoA expression and activity in cultured VSMCs. *n* = 4. **I** Knockdown of RhoA did not block the effects of glucose (30 mM) on phosphorylation of MLC and MYPT1 in cultured VSMCs. *n* = 5. **J** Knockdown of MYPT1 impaired the effects of glucose (30 mM) on MLC phosphorylation in cultured VSMCs. *n* = 5. **K** The effects of TEMPO, mitoTEMPO, and α-CCA pretreatments on glucose influx-induced phosphorylation of MYPT1 and MLC in VSMCs. *n* = 5. Error bars represent SEM. ^*^P < 0.05. ^**^P < 0.01.

To explore the underlying mechanism, we initially tested whether Ca^2+^ signaling is involved in glucose-induced vascular constriction. As shown in Fig. 4A, glucose and Glu-Ins showed little effects on intracellular Ca^2+^ as detected by fluo-4 in cultured VSMCs. In addition, FCCP, NH_4_^+^ and Ch_3_COO^-^ increased intracellular Ca^2+^ levels, while nigericin decreased intracellular Ca^2+^ levels in cultured VSMCs, indicating ΔΨm-regulated VSMC contraction independently of Ca^2+^ signaling (Fig. 4B). Furthermore, Glu-Ins or glucose still promoted VSMC contraction in isolated aortas and cultured VSMCs even when the aortas or VSMCs were pretreated with TG, an endoplasmic reticular Ca^2+^-ATPase inhibitor, or BAPTA-AM, a cell-permeable cytosolic calcium chelator (Fig. 4C, D). These results suggested that glucose-induced VSMC contraction is not likely to be mediated by intracellular Ca^2+^ signaling.

In addition to the Ca^2+^-dependent activation of MLCK, MLCP is the other component in the regulation of VSMC contraction. To evaluate this possibility, we first explored the effects of 2 distinct non-isoform-selective ROCK inhibitors, Y27632 and Fasudil^25^. Both Y27632 and Fasudil inhibited glucose-induced MLC phosphorylation in VSMCs (Fig. 4E). In addition, glucose increased the phosphorylation levels of both MYPT1 and CPI-17 by 92% and 35%, respectively (Fig. 4F), suggesting that glucose inhibits MLCP activity. Consistently, nigericin and NH_4_^+^ increased MYPT1 phosphorylation and FCCP and CH_3_CHOO^-^ decreased MYPT1 phosphorylation (Fig. 4G). However, glucose showed little effect on the expression and activity of RhoA, as well as other molecules of Rho family (Fig. 4H and supplemental Fig. 5). Knockdown of RhoA did not block the effects of glucose on phosphorylation of MLC and MYPT1 (Fig. 4I), suggesting that glucose-induced VSMC contraction is not mediated by RhoA. Although the upstream regulator of MLCP has not been defined, knockdown of MYPT1 impaired the effects of glucose on VSMC contraction (Fig. 4J), suggesting that MYPT1 plays a critical role in regulation of smooth muscle tone by glucose. Since mitochondrial hyperpolarization facilities ROS production and ROS plays an important role in regulating various cellular signals, the role of ROS in glucose influx-induced MYPT1 phosphorylation has been tested. Pretreatment of VSMCs with TEMPO (1 μM), a ROS scavenger, showed no significant effects on glucose-induced MYPT1 phosphorylation, while pretreatment of VSMCs with mitoTEMPO (1 μM), a mitochondrial ROS scavenger, abolished the effects of glucose-induced MYPT1 phosphorylation (Fig. 4K), suggesting that mitochondrial hyperpolarization was not likely to phosphorylate MYPT1 through increasing cytosolic ROS. In addition, α-CCA pretreatment (200 μM) also abolished the effects of glucose-induced MYPT1 phosphorylation. These results suggested that glucose-induced VSMC contraction is mediated by the inactivation of MLCP.

### Smooth muscle-specific KO of MYPT1 impaired the effects of glucose on VSMC contraction

**Fig. 5:**
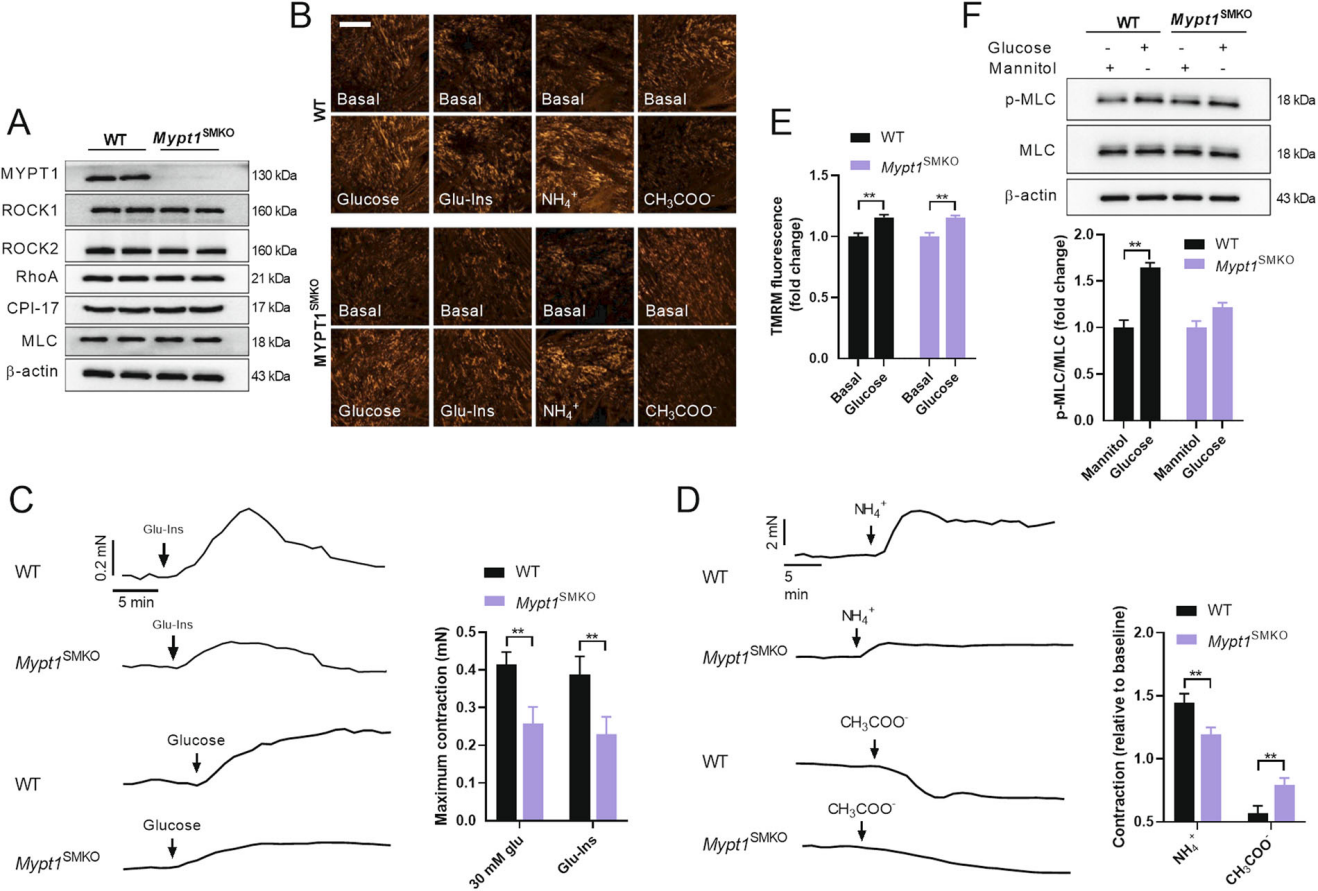
Glucose-induced vascular constriction was impaired in *Mypt1*^SMKO^ mice. **A** Expressions of proteins in MLCP signaling in VSMCs isolated from WT and *Mypt1*^SMKO^ mice. **B** ΔΨ_m_ in response to glucose (30 mM), Glu-Ins, NH_4_^+,^ and Ch_3_COO^-^ in VSMCs in isolated aortas from WT and *Mypt1*^SMKO^ mice. Scale bar, 50 μm. ΔΨ_m_ was detected by TMRM. **C** Vascular tone in response to glucose (30 mM) and Glu-Ins challenges in isolated aortas from WT and *Mypt1*^SMKO^ mice. *n* = 6. **D** Vascular tone in response to NH_4_^+^ and Ch_3_COO^-^ challenges in isolated aortas from WT and *Mypt1*^SMKO^ mice. *n* = 6. **E** ΔΨ_m_ in response to glucose (30 mM) in cultured VSMCs isolated from WT and *Mypt1*^SMKO^ mice. *n* = 5. **F** MLC phosphorylation in response to glucose (30 mM) in cultured VSMCs isolated from WT and *Mypt1*^SMKO^ mice. *n* = 5. Error bars represent SEM. ^*^P < 0.05. ^**^P < 0.01.

To further test the role of MYPT1 in glucose-induced VSMC contraction, a mice model of smooth muscle-specific deletion of MYPT1 (*Mypt1*^SMKO^) were used. Western blots confirmed no detectable MYPT1 expression in thoracic aortas, and MYPT1 KO showed little effect on expressions of other proteins in MLCP signaling (Fig. 5A). VSMC ΔΨm in response to glucose, Glu-Ins, NH_4_^+^, and CH_3_COO^-^ was initially detected, the results displayed no differences between isolated aortas from WT and *Mypt1*^SMKO^ mice (Fig. 5B). However, the vascular constriction was impaired in response to glucose, Glu-Ins and NH_4_^+^, and vascular relaxation was attenuated in response to CH_3_COO^-^, in isolated aortas from *Mypt1*^SMKO^ mice compared with that from WT mice (Fig. 5C, D). Similarly, MYPT1 KO displayed little effects on ΔΨm in response to glucose (Fig. 5E), but decreased MLC phosphorylation in response to glucose in cultured VSMCs (Fig. 5F). These results reinforced the notion that glucose-induced VSMC contraction is mediated by phosphorylation of MYPT1.

### *Mypt1*^SMKO^ mice displayed glucose intolerance and impaired circulating metabolite clearance

**Fig. 6:**
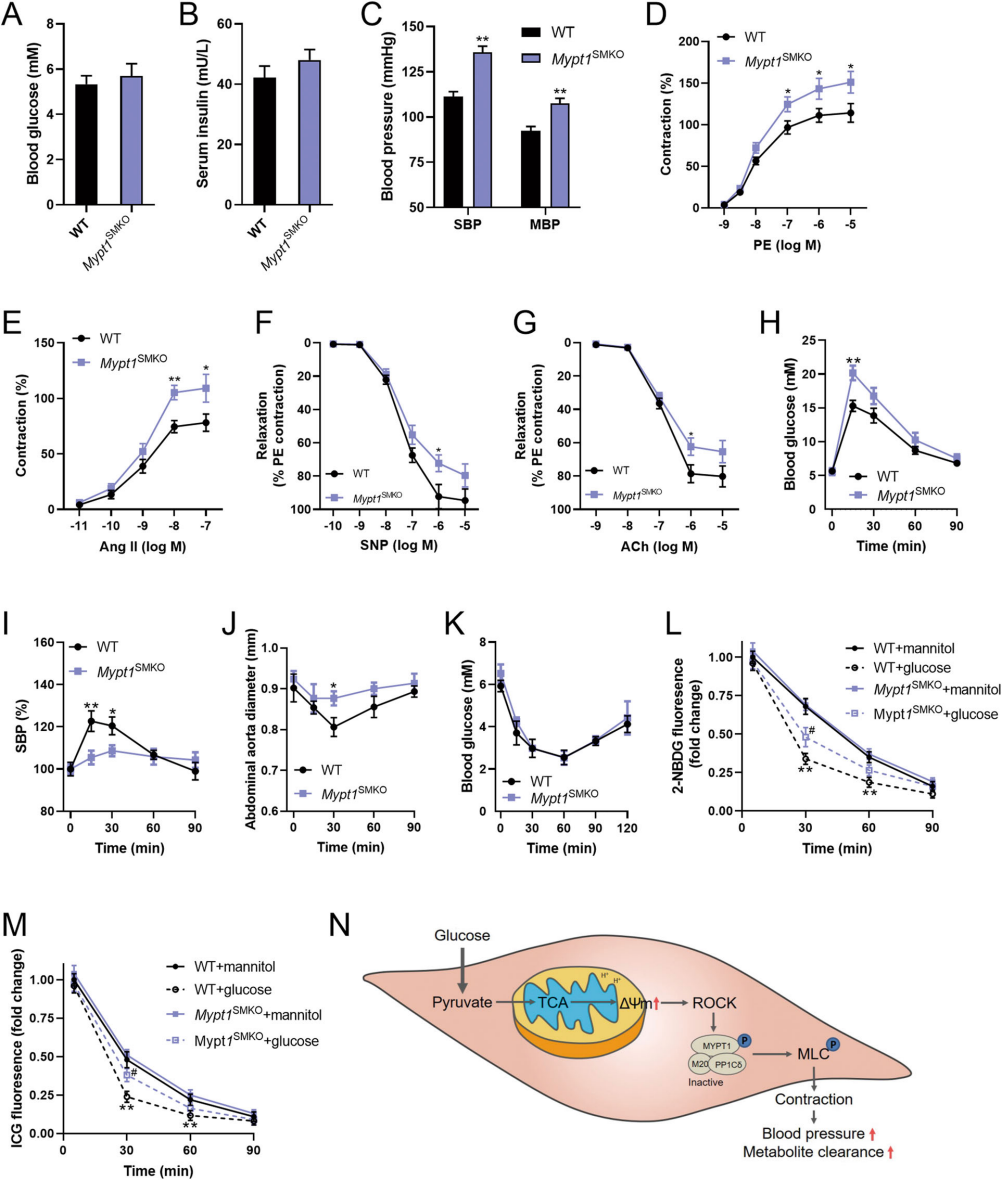
*Mypt1*^SMKO^ mice displayed glucose intolerance and impaired metabolite clearance in response to glucose challenge. **A**, **B** Fasting blood glucose (**A**) and serum insulin (**B**) in WT and *Mypt1*^SMKO^ mice. *n* = 8. **C** Systolic blood pressure (SBP) and mean blood pressure (MBP) in WT and *Mypt1*^SMKO^ mice. *n* = 8. **D**, **E** Vascular constriction in response to PE (**D**) and Angiotensin II (**E**) in isolated aortas from WT and *Mypt1*^SMKO^ mice. *n* = 6. **F**, **G** Vascular relaxation in response to SNP (**F**) and ACh (**G**) in isolated aortas from WT and *Mypt1*^SMKO^ mice. *n* = 6. **H**–**J** Blood glucose (**H**), SBP (**I**), and abdominal aorta diameter (**J**) in response to glucose challenge in WT and *Mypt1*^SMKO^ mice. *n* = 8. **K** Blood glucose in response to insulin challenge in WT and *Mypt1*^SMKO^ mice. *n* = 8. **L**, **M** Circulating 2-NBDG (**L**) and ICG (**M**) fluorescence in response to glucose challenge in WT and *Mypt1*^SMKO^ mice. *n* = 8. ^*^, vs. WT + saline; #, vs. WT + glucose. **N** The graphic summary of our findings. Error bars represent SEM. ^*^P < 0.05. ^#^*P* < 0.05. ^**^P < 0.01.

*Mypt1*^SMKO^ mice displayed normal fasting blood glucose and serum insulin (Fig. 6A, B), but increased systolic and diastolic BP (Fig. 6C), indicating that MYPT1 KO resulted in vascular dysfunction. MYPT1 KO enhanced vascular contraction of isolated aortas in response to PE and Angiotensin II (Fig. 6D, E), and inhibited vascular relaxation in response to SNP and ACh (Fig. 6F, G). Although the blood glucose was increased higher in response to glucose challenge (Fig. 6H), systolic BP and vascular constriction showed little changes in *Mypt1*^SMKO^ mice (Fig. 6I, J). In addition, insulin tolerance was not changed in response to insulin challenge in *Mypt1*^SMKO^ mice (Fig. 6K), suggesting that glucose-induced vascular contraction may be involved in the regulation of metabolite clearance. To test the hypothesis, we used 2-NBDG and ICG to trace the metabolite clearance in circulation. As shown in Fig. 6L, M, both the clearance rates of 2-NBDG and ICG were enhanced by glucose challenge in WT mice, while the effects of glucose challenge on promoting the clearance rates of 2-NBDG and ICG were attenuated in *Mypt1*^SMKO^ mice. These results suggested that glucose-induced vascular constriction contributes to circulating metabolite clearance in mice.

## Discussion

Long-term exposure of the vasculature to metabolic disturbances leaves a persistent imprint on VSMCs, which potentially underlies macrovascular complications in metabolic disorders. However, the physiological effects of metabolic influx on vascular tone regulation remain largely unknown. Here, we found that glucose influx induces vascular constriction through mitochondrial hyperpolarization in VSMCs. Mechanistically, ΔΨm positively phosphorylates MYPT1 which increases MLC phosphorylation through inhibition of MLCP (Fig. 6N). These findings represent a novel mechanism underlying vascular constriction.

Although BP maintains constant as a result of intrinsic cardiovascular regulatory mechanisms, it is characterized by marked fluctuations that have important significance in both physiological and pathological processes^26,27^. For example, a temporary spike in BP in response to stress facilitates blood perfusion to important organs to increase energy supply. Here we showed that glucose influx induces temporary spikes in vascular constriction and BP, which facilitate organ perfusion and metabolite clearance in circulation. A recent study also showed that glucose influx increased vascular tone in cerebral arteries^28^. In addition, we found that glucose challenge promoted clearance of glucose, 2-NBDG, and ICG in circulation, and these responses were attenuated in Mypt1^SMKO^ mice, indicating that blood glucose fluctuation plays a physiological role in the regulation of circulating metabolite homeostasis. Transient increase in blood glucose is observed in various physiological conditions, such as feeding behavior and acute stress. A temporary spike in BP in response to acute stress has been well-documented, while the observations of postprandial BP are not consistent. Both postprandial hypertension and postprandial hypotension have been reported, but most of these data are collected in aging or patients^29–31^. As the metabolic disturbance following feeding is more complex than the glucose challenge, changes in postprandial BP in healthy individuals need to be further investigated.

Different from striated muscle cells, VSMCs exhibit unusually high rates of glycolysis^11^. In spite of the low contribution of mitochondria to VSMC bioenergetics, evidence has shown that mitochondria widely regulate VSMC phenotype and function^32–34^. Our results extended these findings that mitochondrial oxidation promotes VSMC contraction. Interestingly, the process is not dependent on mitochondria-derived ATP, but dependent on ΔΨm which positively regulates VSMC contraction as evidenced by a series of experiments. In fact, studies have shown that mitochondrion is the most sophisticated and dynamic responsive sensing system and depolarization or hyperpolarization of mitochondria plays a critical role in the regulation of various signals or processes, including reactive oxygen species, mitochondrial permeability, Ca^2+^, antiviral immunity, apoptosis, and aging^35^. Depolarization of ΔΨm is also a basic characteristic of several mitochondrial events including mitochondrial flash, flicker, and oscillation, which serve as important signals in multiple physiological and pathological processes^36–38^. We found that mitochondrial hyperpolarization mediates the glucose influx-induced VSMC contraction, and inhibition of ΔΨm fluctuation blocked the effects of glucose influx on the promotion of vascular constriction.

Smooth muscle tone is largely dependent on the relative activities of MLCK and MLCP^39^. As glucose influx has little effects on intracellular Ca^2+^ concentration and inhibition of intracellular Ca^2+^ fluctuation did not block the effects of glucose influx, glucose influx-induced VSMC contraction is not likely to be MLCK-mediated. Here, we found that glucose influx inhibited MLCP activity. Multiple signals have been reported to regulate MLCP activity in VSMCs^39^, among which ROCK is the major player. Glucose influx activated ROCK signaling and inhibition of ROCK signaling blocked the effects of glucose influx on VSMC contraction, indicating ROCK activation as a major step in ΔΨm-regulated smooth muscle tone. However, as the key molecule which activates ROCK signaling, RhoA is not involved in glucose influx-induced VSMC contraction as evidenced by the fact that glucose influx did not activate RhoA, and RhoA knockdown showed little effects on glucose influx-induced VSMC contraction. These results suggested that other small GTPases may be involved in the process. In fact, other Rho family proteins, including proteins from the Rho, Cdc42, Rac, and Rnd subfamilies, and even proteins from Ras superfamily of monomeric GTPases have been reported to be involved in the regulation of ROCK activity^39,40^, which should be tested in the future.

MLCP is mainly regulated by MYPT1 and CPI-17. Our data showed that both MYPT1 and CPI-17 are activated by mitochondrial hyperpolarization, in which MYPT1 is more responsive. Apart from protein kinase C, CPI-17 can also be activated by ROCK signaling^41^. MYPT1 is identified as an essential protein in the smooth muscle myosin phosphorylation module. MYPT1 enhances the catalytic activity and specificity of PP1cδ toward phosphorylated MLC in its binding to myosin. PP1cδ activity was inhibited by ROCK-induced phosphorylation of MYPT1 at Thr-696 or Thr-853, which has been shown to be either Ca^2+^-sensitive or Ca^2+^-independent^17^. Our data showed that ΔΨm-regulated MYPT1 activity was Ca^2+^-independent as evidenced by that modulation of Ca^2+^ signaling showed little effects on ΔΨm-regulated MYPT1 activity. Although mounting evidence showed that MYPT1 played a central role in regulating MLCP activity and the phenotypic changes associated with adult Mypt1^SMKO^ mice were modest, suggesting that MYPT1 is more likely to be a regulator of smooth muscle tone^18,20^. Here, we showed that knockout of MYPT1 in smooth muscle cells largely abolished the effects of both glucose and ΔΨm-regulated VSMC contraction in vivo and in vitro, indicating that MYPT1 plays a critical role in glucose influx-regulated VSMC contraction. Our results showed that besides MYPT1, there must be other regulators such as CPI-17 in the process. However, how ΔΨm affects phosphorylation of MYPT1 has not been identified in the study. We tested the role of ROS in glucose influx-induced MYPT1 phosphorylation and found that cytosolic ROS was not likely to mediate ΔΨm-regulated cytosol protein phosphorylation, but mitochondrial ROS was involved in ΔΨm-regulated MYPT1 phosphorylation, suggesting that mitochondrial ROS may not need to be converted as cytosolic ROS to regulate cytosol protein phosphorylation. The underlying mechanism in mitochondrial ROS-regulated MYPT1 phosphorylation should be investigated in the future.

Taken together, our findings demonstrated that glucose-induced VSMC contraction is mediated by mitochondrial hyperpolarization-activated MYPT1. ΔΨm positively regulates MYPT1 activity which inhibits MLCP activity. This finding represents a novel mechanism underlying metabolic influx-induced vascular constriction and suggests that physiological blood glucose elevation contributes to physiological BP control and circulating metabolite clearance.

## Supplementary information

SUPPLEMENTAL MATERIAL

## References

[1] Arnett DK, Claas SA (2018). Omics of blood pressure and hypertension. Circ. Res.

[2] Padmanabhan S, Joe B (2017). Towards precision medicine for hypertension: a review of genomic, epigenomic, and microbiomic effects on blood pressure in experimental rat models and humans. Physiol. Rev..

[3] Saxton SN, Clark BJ, Withers SB, Eringa EC, Heagerty AM (2019). Mechanistic links between obesity, diabetes, and blood pressure: role of perivascular adipose tissue. Physiol. Rev..

[4] Webb RC (2003). Smooth muscle contraction and relaxation. Adv. Physiol. Educ..

[5] Brozovich FV, Nicholson CJ, Degen CV, Gao YZ, Aggarwal M, Morgan KG (2016). Mechanisms of vascular smooth muscle contraction and the basis for pharmacologic treatment of smooth muscle disorders. Pharm. Rev..

[6] Ford TJ, Corcoran D, Padmanabhan S, Aman A, Rocchiccioli P, Good R (2020). Genetic dysregulation of endothelin-1 is implicated in coronary microvascular dysfunction. Eur. Heart J..

[7] Sutendra G, Bonnet S, Rochefort G, Haromy A, Folmes KD, Lopaschuk GD (2010). Fatty acid oxidation and malonyl-CoA decarboxylase in the vascular remodeling of pulmonary hypertension. Sci. Transl. Med..

[8] Guo Y, Wang S, Liu Y, Fan L, Booz GW, Roman RJ (2020). Accelerated cerebral vascular injury in diabetes is associated with vascular smooth muscle cell dysfunction. Geroscience.

[9] Wang DD, Toledo E, Hruby A, Rosner BA, Willett WC, Sun Q (2017). Plasma ceramides, mediterranean diet, and incident cardiovascular disease in the PREDIMED Trial (Prevencion con Dieta Mediterranea). Circulation.

[10] Dunham-Snary KJ, Wu D, Potus F, Sykes EA, Mewburn JD, Charles RL (2019). Ndufs2, a core subunit of mitochondrial complex i, is essential for acute oxygen-sensing and hypoxic pulmonary vasoconstriction. Circ. Res..

[11] Butler TM, Siegman MJ (1985). High-energy phosphate metabolism in vascular smooth muscle. Annu. Rev. Physiol..

[12] Paul RJ (1983). Functional compartmentalization of oxidative and glycolytic metabolism in vascular smooth muscle. Am. J. Physiol..

[13] Chiong M, Cartes-Saavedra B, Norambuena-Soto I, Mondaca-Ruff D, Morales PE, Garcia-Miguel M (2014). Mitochondrial metabolism and the control of vascular smooth muscle cell proliferation. Front Cell Dev. Biol..

[14] Chalmers S, Saunter C, Wilson C, Coats P, Girkin JM, McCarron JG (2012). Mitochondrial motility and vascular smooth muscle proliferation. Arterioscler Thromb. Vasc. Biol..

[15] Salabei JK, Hill BG (2013). Mitochondrial fission induced by platelet-derived growth factor regulates vascular smooth muscle cell bioenergetics and cell proliferation. Redox Biol..

[16] Lincoln TM (2007). Myosin phosphatase regulatory pathways: different functions or redundant functions?. Circ. Res..

[17] Dimopoulos GJ, Semba S, Kitazawa K, Eto M, Kitazawa T (2007). Ca^2+^-dependent rapid Ca^2+^ sensitization of contraction in arterial smooth muscle. Circ. Res.

[18] Qiao YN, He WQ, Chen CP, Zhang CH, Zhao W, Wang P (2014). Myosin phosphatase target subunit 1 (MYPT1) regulates the contraction and relaxation of vascular smooth muscle and maintains blood pressure. J. Biol. Chem..

[19] Huang QF, Aparicio LS, Thijs L, Wei FF, Melgarejo JD, Cheng YB (2020). Cardiovascular end points and mortality are not closer associated with central than peripheral pulsatile blood pressure components. Hypertension.

[20] He WQ, Qiao YN, Peng YJ, Zha JM, Zhang CH, Chen C (2013). Altered contractile phenotypes of intestinal smooth muscle in mice deficient in myosin phosphatase target subunit 1. Gastroenterology.

[21] Yang L, Zhang J, Xing W, Zhang X, Xu J, Zhang H (2016). SIRT3 deficiency induces endothelial insulin resistance and blunts endothelial-dependent vasorelaxation in mice and human with obesity. Sci. Rep..

[22] Yang F, Chen Q, He S, Yang M, Maguire EM, An W (2018). miR-22 is a novel mediator of vascular smooth muscle cell phenotypic modulation and neointima formation. Circulation.

[23] Zhang X, Xu J, Cai X, Ji L, Li J, Cao B (2014). Acute insulin resistance mediated by advanced glycation endproducts in severely burned rats. Crit. Care Med.

[24] Johnson LV, Walsh ML, Bockus BJ, Chen LB (1981). Monitoring of relative mitochondrial membrane potential in living cells by fluorescence microscopy. J. Cell Biol..

[25] Liao JK, Seto M, Noma K (2007). Rho kinase (ROCK) inhibitors. J. Cardiovasc Pharm..

[26] Wright BJ, O’Brien S, Hazi A, Kent S (2014). Increased systolic blood pressure reactivity to acute stress is related with better self-reported health. Sci. Rep..

[27] Ayada C, Toru U, Korkut Y (2015). The relationship of stress and blood pressure effectors. Hippokratia.

[28] Syed AU, Reddy GR, Ghosh D, Prada MP, Nystoriak MA, Morotti S (2019). Adenylyl cyclase 5-generated cAMP controls cerebral vascular reactivity during diabetic hyperglycemia. J. Clin. Invest..

[29] Ward KA, DiPette DJ, Held TN, Jain RK (1991). Effect of intravenous versus intraperitoneal glucose injection on systemic hemodynamics and blood flow rate in normal and tumor tissues in rats. Cancer Res..

[30] Huber DA, Carmo JM, Castania JA, Fazan R, Salgado HC (2007). Does acute hyperglycemia alter rat aortic depressor nerve function?. Braz. J. Med Biol. Res..

[31] Uetani E, Tabara Y, Igase M, Guo H, Kido T, Ochi N (2012). Postprandial hypertension, an overlooked risk marker for arteriosclerosis. Atherosclerosis.

[32] Yu EP, Bennett MR (2014). Mitochondrial DNA damage and atherosclerosis. Trends Endocrinol. Metab..

[33] Dasgupta A, Wu D, Tian L, Xiong PY, Dunham-Snary KJ, Chen KH (2020). Mitochondria in the pulmonary vasculature in health and disease: oxygen-sensing, metabolism, and dynamics. Compr. Physiol..

[34] Liu YF, Zhu JJ, Yu Tian X, Liu H, Zhang T, Zhang YP (2020). Hypermethylation of mitochondrial DNA in vascular smooth muscle cells impairs cell contractility. Cell Death Dis..

[35] Dromparis P, Michelakis ED (2013). Mitochondria in vascular health and disease. Annu Rev. Physiol..

[36] Wang X, Zhang X, Huang Z, Wu D, Liu B, Zhang R (2016). Protons trigger mitochondrial flashes. Biophys. J..

[37] Chalmers S, Saunter CD, Girkin JM, McCarron JG (2015). Flicker-assisted localization microscopy reveals altered mitochondrial architecture in hypertension. Sci. Rep..

[38] Chalmers S, McCarron JG (2008). The mitochondrial membrane potential and Ca^2+^ oscillations in smooth muscle. J. Cell Sci..

[39] Puetz S, Lubomirov LT, Pfitzer G (2009). Regulation of smooth muscle contraction by small GTPases. Physiology (Bethesda).

[40] Etienne-Manneville S, Hall A (2002). Rho GTPases in cell biology. Nature.

[41] Koyama M, Ito M, Feng J, Seko T, Shiraki K, Takase K (2000). Phosphorylation of CPI-17, an inhibitory phosphoprotein of smooth muscle myosin phosphatase, by Rho-kinase. FEBS Lett..

