# Supplementary material for: Acute glucose influx-induced mitochondrial hyperpolarization inactivates myosin phosphatase as a novel mechanism of vascular smooth muscle contraction
Source: Cell Death Dis. 2021 Feb 12;12(2):176. doi: 10.1038/s41419-021-03462-9 (PMC7881016; doi:10.1038/s41419-021-03462-9)
Supplement: Supplementary file 1 — SUPPLEMENTAL MATERIAL [file 41419_2021_3462_MOESM1_ESM.docx]

**DATA SUPPLEMENT**

**Acute glucose influx-induced mitochondrial hyperpolarization inactivates myosin phosphatase as a novel mechanism of vascular smooth muscle contraction**

Jie Xu^1, 2^, Hongyan Yang^1^, Lu Yang^3^, Zhen Wang^1^, Xinghua Qin^1^, Jiaheng Zhou^1^, Ling Dong^1^, Jia Li^1^, Minsheng Zhu^4^, Xing Zhang^1,*^, Feng Gao^1^

^1^ School of Aerospace Medicine, Fourth Military Medical University, Xi’an 710032, China. ^2^ Department of Cardiology, 986^th^ Hospital, Fourth Military Medical University, Xi’an 710032, China. ^3^ School of Basic Medical Sciences, Fourth Military Medical University, Xi’an 710032, China. ^4^ Model Animal Research Center, Nanjing University, Nanjing 210061, China.

**^*^Correspondence should be addressed to:**

Xing Zhang, Ph.D., School of Aerospace Medicine, Fourth Military Medical University, Xi’an 710032, China. Tel: 86-29-84711275. E-mail: [zhangxing@fmmu.edu.cn](mailto:zhangxing@fmmu.edu.cn)

**Table 1. Characteristics of the subjects involved**

| **Characteristics** | **Mean ± SEM** |
| --- | --- |
|  |  |
| **Subjects No.** | 8 |
| **Age (years)** | 30.4±1.2 |
| **Weight (kg)** | 71.0±1.9 |
| **Height (cm)** | 177.1±2.8 |
| **BMI (kg/m^2^)** | 22.7±0.7 |

**Table 2. siRNA sequences**

| **siRNA (No.)** | **Sequence (5’-3’)** |
| --- | --- |
| RhoA siRNA 1 | 5′-AUGGAAAGCAGGUAGAGUU-3′ |
| RhoA siRNA 2 | 5′-GAACUAUGUGGCAGAUAUC-3′ |
| MYPT1 siRNA | 5′-GAACGAGACUUGCGUAUGUUU-3′ |
| Negative control | 5′-AUGAACGUGAAUUGCUCAA-3′ |


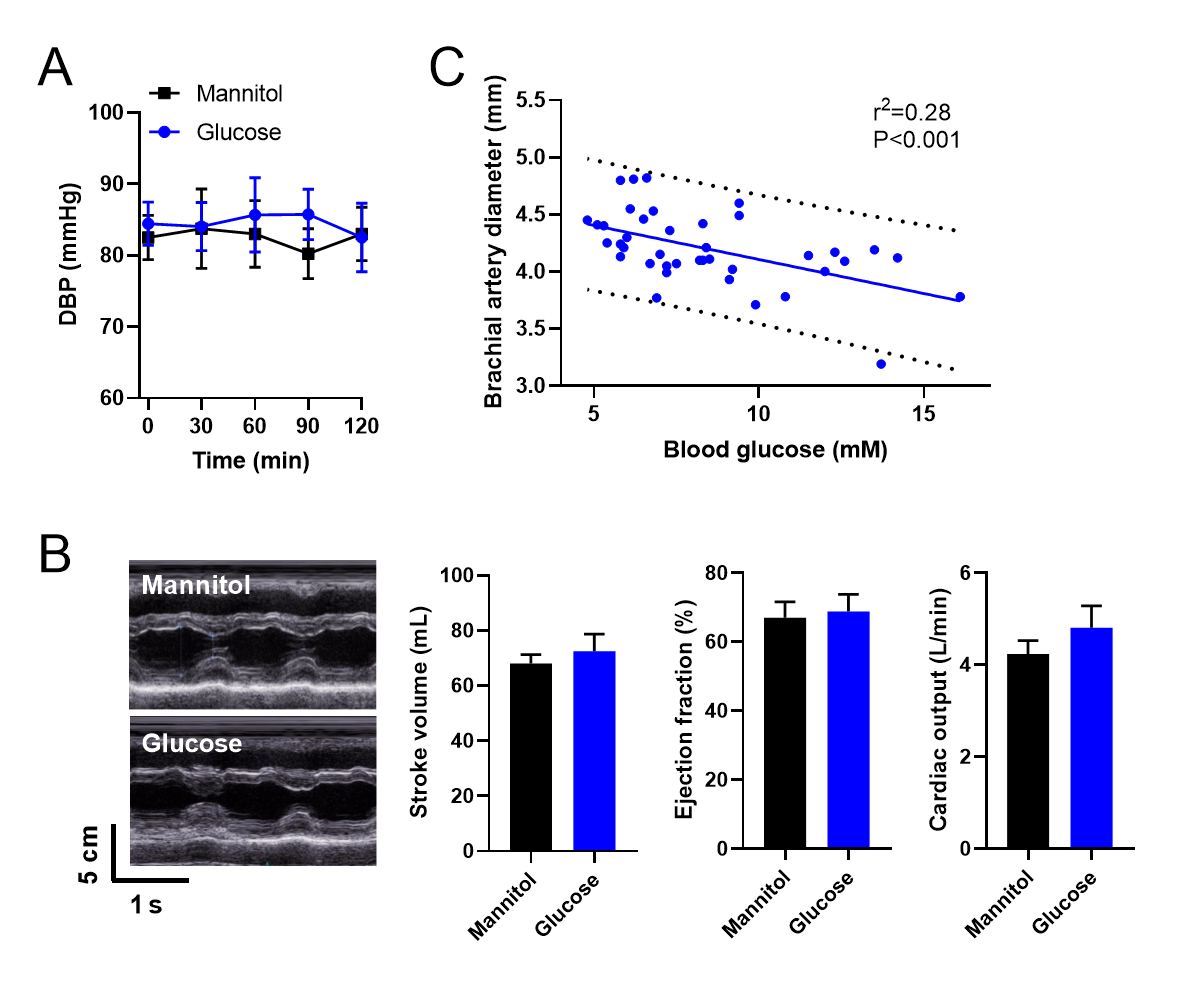


**Figure 1. Diastolic blood pressure (DBP) and cardiac function post glucose challenge in humans.**

A. DBP in response to oral glucose challenge in humans. B. Cardiac function in response to oral glucose challenge in humans. Representative images of cardiac function during glucose challenge are shown in left, and the quantified results are shown in right. C. A negative linear relation between blood glucose and the maximal brachial artery diameter in subjects with glucose challenge. n=8 subjects.


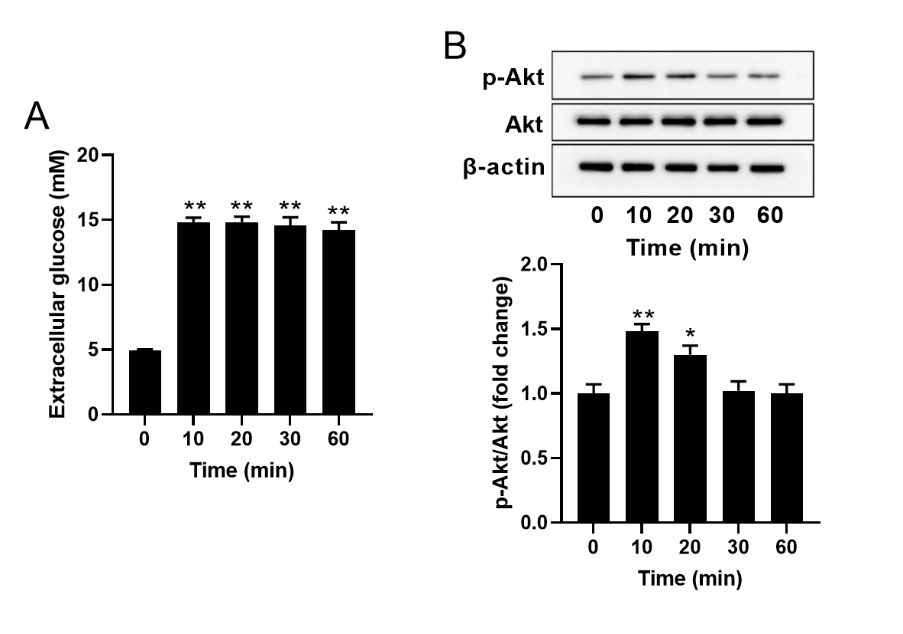


**Figure 2. A transient response of insulin in VSMCs with Glu-Ins treatment.**

A. Extracellular glucose levels in incubation medium of isolated thoracic aortas in response to Glu-Ins treatment. B. Akt and its phosphorylation in isolated thoracic aortas in response to Glu-Ins treatment. n=6. *, P<0.05; **, P<0.01; vs. 0.


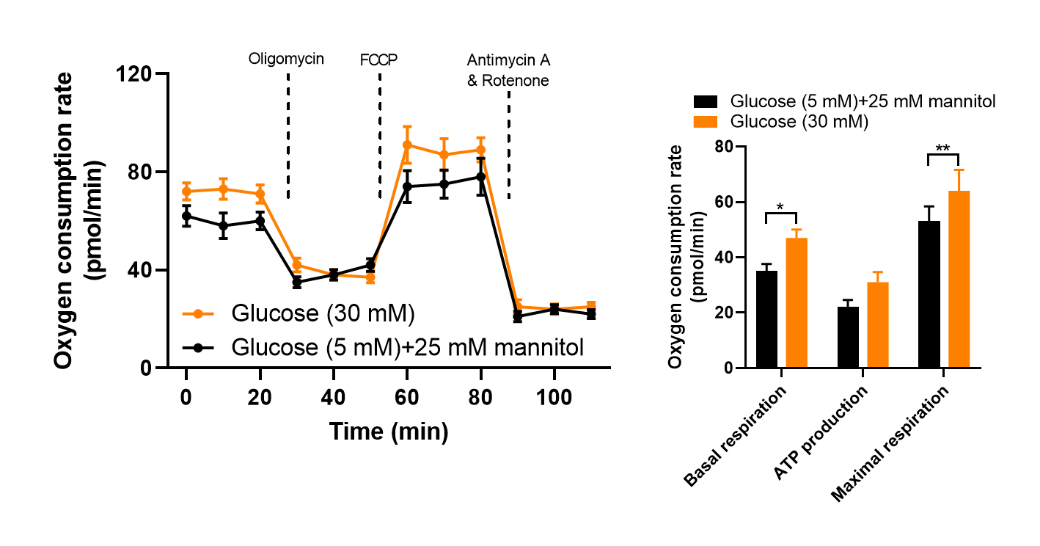


**Figure 3. Glucose (30 mM) increased mitochondrial respiration in cultured VSMCs.**

VSMCs were cultured in XF24 microplates (Seahorse Bioscience). The cells were incubated in Tyrode’s solution with either 5.0 mM glucose or 30 mM glucose. VSMC respiration was measured in an XF24 Extracellular Flux Analyzer (Seahorse Bioscience) with the injection of oligomycin (1 μM), FCCP (1 μM), and rotenone (1 μM) and antimycin A (1 μM) sequentially. Basal respiration, respiration for ATP production and maximal respiration were calculated following the manufacturer’s instructions. n=5. *, P<0.05; **, P<0.01.


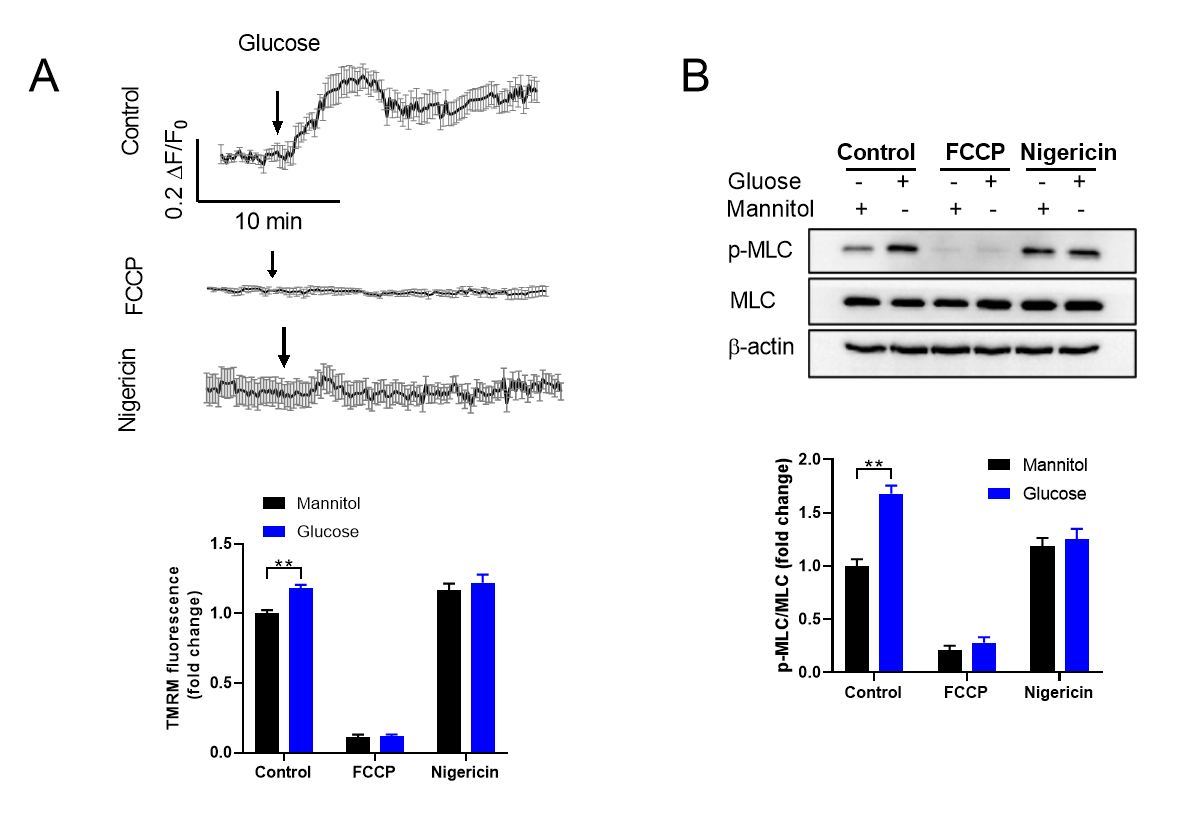


**Figure 4. Mitochondrial hyperpolarization contributes to glucose-induced vascular constriction in VSMCs**

A. Glucose (30 mM)-induced mitochondrial hyperpolarization was blocked by preincubation of cultured VSMCs with FCCP or nigericin. ΔΨ_m_ was detected by TMRM. n=5. B. Preincubation of cultured VSMCs with FCCP or nigericin inhibited glucose (30 mM)-induced MLC phosphorylation in cultured VSMCs. Typical results were shown in up, and the quantified results were shown in down. n=5. **, P<0.01.


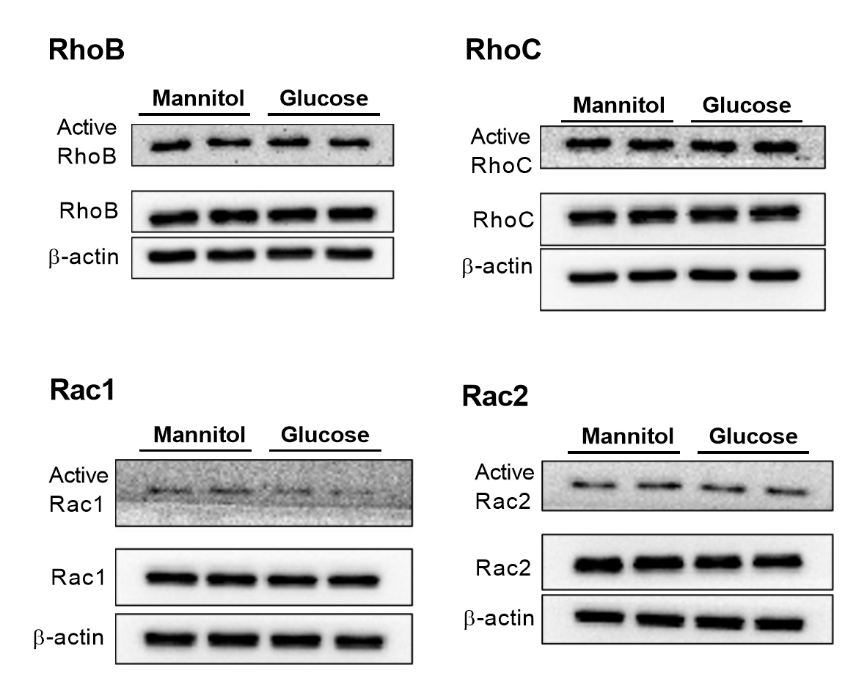


**Figure 5. RhoB, RhoC, Rac1 and Rac2** **expressions and activities in response to glucose challenge in cultured VSMCs.**

Glucose showed little effects on expressions and activities of RhoB, RhoC, Rac1, and Rac2. The activities were detected by assay kits from Cell Biolabs (STA-401-1, STA-401-2, STA-403-B and STA-403-C).
